# Cell death-based treatment of neuroblastoma

**DOI:** 10.1038/s41419-017-0060-1

**Published:** 2018-01-25

**Authors:** Kadri Valter, Boris Zhivotovsky, Vladimir Gogvadze

**Affiliations:** 1 Division of Toxicology, Institute of Environmental Medicine, Karolinska Institutet, Box 210, Stockholm, SE-171 77 Sweden; 2 MV Lomonosov Moscow State University, Moscow, 119991 Russia

## Abstract

Neuroblastoma (NB) is the most common solid childhood tumor outside the brain and causes 15% of childhood cancer-related mortality. The main drivers of NB formation are neural crest cell-derived sympathoadrenal cells that undergo abnormal genetic arrangements. Moreover, NB is a complex disease that has high heterogeneity and is therefore difficult to target for successful therapy. Thus, a better understanding of NB development helps to improve treatment and increase the survival rate. One of the major causes of sporadic NB is known to be *MYCN* amplification and mutations in *ALK* (anaplastic lymphoma kinase) are responsible for familial NB. Many other genetic abnormalities can be found; however, they are not considered as driver mutations, rather they support tumor aggressiveness. Tumor cell elimination via cell death is widely accepted as a successful technique. Therefore, in this review, we provide a thorough overview of how different modes of cell death and treatment strategies, such as immunotherapy or spontaneous regression, are or can be applied for NB elimination. In addition, several currently used and innovative approaches and their suitability for clinical testing and usage will be discussed. Moreover, significant attention will be given to combined therapies that show more effective results with fewer side effects than drugs targeting only one specific protein or pathway.

## Introduction

Neuroblastoma (NB) is the most common solid childhood tumor outside the brain. It originates from primitive cells of the sympathetic nervous system^1^. NB causes 15% of childhood cancer-related mortality and overall survival rate for metastatic tumors is considerably low, 40% after 5 years^2,3^. Most incidences are diagnosed during the first year of life, which also gives a better prospect for the outcome, whereas older patients have a poorer diagnosis^4,5^. In some NB cases, spontaneous regression has also been detected; however, underlying mechanisms remain unclear^6,7^. Moreover, NB is a complex disease that has high genetic, biological, clinical, and morphological heterogeneity, and is therefore difficult to target for successful therapy^8–10^. Thus, NB is under thorough investigation to better understand its progression and to improve the treatment to increase the survival rate.

Several classification systems have been used in order to improve risk assessment and prognosis of NB. For example, the outcome of the disease can be assessed by the presence or absence of stroma, the degree of differentiation, and the mitosis-karyorrhexis index^11^. Currently, even more parameters are used for the classification of NBs, such as stage, age, histologic category, grade of tumor differentiation, the status of the MYCN oncogene, chromosome 11q status, and DNA ploidy. These are the most statistically significant and clinically relevant factors  in use to describe two stages of localized (L1 and L2) and two stages of metastatic disease (M and MS)^12^.

**Fig. 1:**
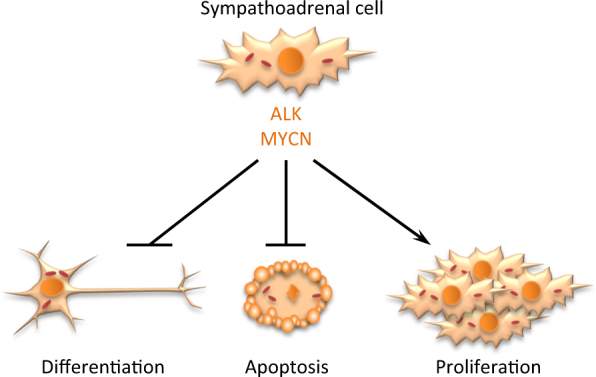
The main drivers of NB formation are neural crest cell-derived sympathoadrenal cells with genetic abnormalities. Several germline and sporadic genomic rearrangements have been detected in NB, for example, in *ALK* (anaplastic lymphoma kinase) and *MYCN* genes. These changes are responsible for the suppression of differentiation and apoptosis to support rapid proliferation of the cells

The main drivers of NB formation are abnormalities in sympathoadrenal cells that derive from neural crest cells (Figure 1)^13^. Several germline and sporadic genomic rearrangements have been detected in NB, for example, *LIN28B* (encoding lin 28 homolog B)^14^, *PHOX2B* (paired-like homeobox 2b)^15^, *ALK* (anaplastic lymphoma kinase)^16^, *GALNT14* (polypeptide *N*-acetylgalactosaminyltransferase 14)^17^, and *MYCN*^18^ (Table 1). Around 2% of NB cases appear to be hereditary, with *ALK* being the first gene identified to be responsible for familial NB^16,19^. Furthermore, *MYCN* oncogene amplification is found in 20% of all NB cases, especially in patients who are resistant to therapy and have poor prognosis^18,20,21^. More than 50% of these high-risk patients relapse even after intensive treatment^22^. Whole-genome sequencing has been used to identify additional mutations and genes responsible for *de novo* NB development, but no other specific “NB driver mutations” have been found^23,24^. Thus, *MYCN* amplification seems to be the major cause of sporadic NB and other mutations support tumor aggressiveness^25^. Therefore, investigation of the *MYCN* gene amplification is considered to be a mandatory step for treatment specification^26^.

**Table 1:** Frequency of germline and sporadic genomic rearrangements in NB

Gene or region	Function	Modification	Frequency (%)	Reference
ALK	Receptor tyrosine kinase	Mutation Amplification	1	^16^
MYCN	Transcription factor	Overexpression Amplification	20	^18,41,42^
LIN28B	Suppressor of miRNA biogenesis	Overexpression Amplification	NA	^14,57^
TERT	Telomerase reverse transcriptase	Rearrengements	25	^58,59^
ATRX	RNA helicase	Rearrengements	10	^23^
17q	NA	Gain	50	^60^
1p36	NA	Deletion	30	^61^
11q1	NA	Deletion	30	^61^

In this review, we provide a thorough overview of how different modes of cell death are exploited or can be employed as treatment for NB. In addition, several novel or already clinically tested drugs against NB and their mechanisms of action are discussed. A special emphasis is also placed on combined therapies that attack many pathways and have been shown to be more effective than drugs targeting only one specific protein or pathway.

## Genetic background

### Anaplastic lymphoma kinase

Changes in the *ALK* gene are identified as being responsible for ~ 50% of familial and ~ 1% of all NBs^16^ (Table 1). ALK is a member of the insulin receptor superfamily of transmembrane RTKs (receptor tyrosine kinase). Mutations and amplifications of the *ALK* gene can lead to a constitutive activation of ALK that supports cell survival and proliferation in the peripheral neuronal and central nervous system. This can be achieved by the engagement of several pathways, such as Janus kinase–signal transducer and activator of transcription^27^, PI3K–AKT^27^ in anaplastic large cell lymphoma, and/or RAS–mitogen-activated protein kinase^28^ in NB.

The central role of the ALK in NB development makes it a possible target for NB treatment. For example, NB cell lines with constitutively active or overexpressed ALK are susceptible to RNAi and ALK inhibitors^29^. For instance, crizotinib^30^ and entrectinib^31^ reduce the cells’ proliferation rate and are currently in Phase 1/2 trials (NCT00939770, NCT01606878, and NCT02650401) for relapsed or refractory NB; however, there are problems with their off-target effects and acquired resistance. Therefore, new-generation ALK inhibitors are already been developed and tested for NB therapy, for example, lorlatinib (NCT03107988)^32^, AZD3463 (ref. 33), and ceritinib (NCT01742286)^34^. In addition to reducing the proliferation rate, clinical tests have shown that most ALK inhibitors also sensitize NB cells to conventional cytotoxic drugs and their combined use is causing more prominent cell death^35,36^. On the other hand, this approach is helpful for only ALK-positive tumors and, due to the high heterogeneity of NB, more strategies are needed for successful treatment of NBs carrying other mutations.

### MYCN

MYCN is part of the MYC family of transcription factors that regulate several cellular processes including proliferation, cell cycle, glycolysis, glutaminolysis, mitochondrial function, and biogenesis^37–39^. MYCN expression is essential for normal prenatal development and is present until a few weeks after birth^40^. Amplifications of the *MYCN* gene are known to be responsible for increased tumor growth, proliferation, and NB development (Table 1)^41,42^. Deregulation of *MYC* induces cell proliferation and apoptosis; however, this apoptotic signal is inhibited by reducing p53 activity, overexpressing anti-apoptotic proteins, or downregulating pro-apoptotic proteins^43,44^. Thus, a combined suppression of *MYC*-induced apoptosis and *MYC*-driven proliferative signals supports extensive tumor development.

MYCN usually has a very short half-life, but after amplification it is highly expressed and forms heterodimers with MAX to act as a transcriptional factor and support constant NB tumor growth^45^. Therefore, downregulation of MYCN is one possible approach to induce apoptosis, decrease NB proliferation, and/or induce neuronal differentiation^46^. For example, antisense oligonucleotides^47^ and RNAi^48–50^ have been successfully used for MYCN downregulation in NB that resulted in decreased tumor growth, cellular migration, and invasion. The described approach has proved to be effective in the laboratory; however, off-target effects and clinical delivery of these compounds to the tumor site are still problematic.

Blocking the MYCN/MAX interaction is another option for NB therapy, because unbound MAX homodimerizes and stimulates differentiation^51^. Several compounds blocking the heterodimerization, such as 10058-F4 (ref. 52,53) and 10074-G5 (ref. 52), have shown cell cycle arrest, apoptosis, and differentiation *in vitro*, and also increased survival in *MYCN* transgenic mice. Another approach is to inhibit bromodomain and extra-terminal domain family of transcription-regulating proteins by small molecules such as JQ1 (ref. 54), OTX015 (ref. 55), or I-BET762 (ref. 56), which lead to the suppression of *MYCN* transcription and proliferation. These compounds can help high-risk patients with *MYCN*-driven NB; however, thorough clinical testing is still needed. The role of *ALK* and *MYCN* in regulation of NB cell fate is shown on Figure 1.

## Other genomic abnormalities

Overexpression and amplifications of *LIN28B* are very common in NB cells and can in turn lead to high MYCN expression (Table 1)^14,57^. Moreover, whole-genome sequencing revealed that 25% of the patients have rearrangements in *TERT* (encoding telomerase reverse transcriptase)^58,59^ promoter and 10% in transcriptional regulator *ATRX* (encoding the RNA helicase)^23^, supporting rapid cellular proliferation (Table 1). Chromosomal copy number alterations are also represented in almost all NBs, for example, more than 50% have gain of 17q (ref. 60) and 30% have loss of 1p36 and/or 11q1 (ref. 61) (Table 1). These arrangements have a strong correlation with *MYCN* amplification and poor prognosis. However, the function of these regions and how they regulate NB formation is still unclear^60,61^.

## Targeting NB via stimulation of various modes of cell death

### Apoptosis induction in NB therapy

Apoptosis is essential for the normal growth of an organism, being involved in early embryonic and immune system development. It also has an important role in the maintenance of normal tissue homeostasis and helps to eliminate damaged and harmful cells^62^. Therefore, misregulation of apoptotic pathways has an important role in cancer development, because mutations or amplifications in the oncogenes (e.g., *MYC*) can compromise apoptotic pathways. On the other hand, apoptosis induction is the most prominent anticancer strategy.

#### Targeting p53/MDM2 interaction

**Fig. 2:**
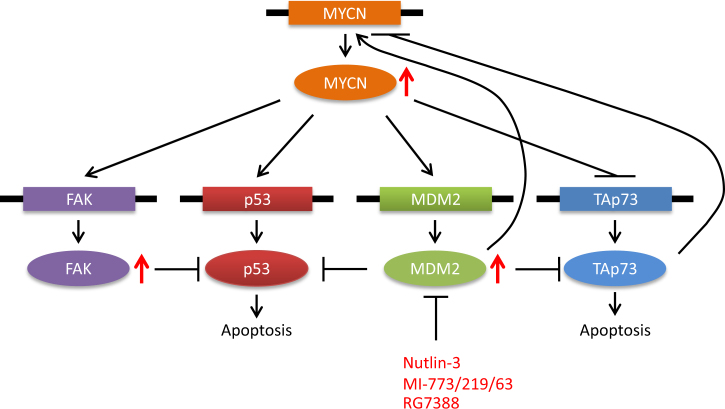
NB cell death can be avoided by overexpression or amplification of MDM2 (murine double minute 2), negative regulator of the p53. The p53–MDM2 pathway may be targeted by small antagonistic molecules, such as nutlin-3, MI-773/219/63, idasanutlin (RG7388), which bind to MDM2, to block its interaction with p53. MYCN also facilitates expression of p53 and FAK (focal adhesion kinase) that in turn interacts with p53 and causes its sequestering in the cytoplasm. Furthermore, MYCN-upregulated MDM2 can in turn bind to *MYCN* and tumor suppressor TAp73, and decrease its transcription to support resistance to the treatment. MYCN and TAp73 might also directly decrease each other expression and influence the outcome of the NB development and treatment

The members of the p53 protein family are important regulators of cell cycle and apoptosis in normal and transformed cells^63^. In addition, p53 as well as p73 act as tumor suppressors. Mutations in the *p53* gene that control cell fate occur in more than 80% of tumor cell lines and more than 40% of human cancers^64^. However, abnormalities of p53 are mostly found in relapsed NB after chemotherapy, but not at the time of the diagnosis^65,66^. Instead, overexpressed MYCN regulates *p53* and *MDM2* (murine double minute 2) expression to achieve stringent control over cell death (Figure 2)^67,68^. Tumors such as NB, which generally have wild-type p53, are likely to induce the degradation of p53 and avoid cell death by overexpression or amplification of MDM2, which is a negative regulator and the primary E3 ubiquitin ligase for p53 (ref. 65,67,69). For instance, MYCN binds to the promoter of *MDM2* to induce its expression and *vice versa*, suggesting that downregulation of MDM2 can also be used to decrease MYCN expression and stabilize p53 to induce apoptosis (Figure 2)^67,70,71^.

Understanding these peculiarities of NB and targeting the p53–MDM2 pathway may be helpful in finding better therapeutic treatments for pediatric patients with wild-type p53 (ref. 72,73). For example, small antagonistic molecules, like nutlin-3 (ref. 74–76), MI-773/219/63 (ref. 75), and idasanutlin (RG7388)^77^, which bind to MDM2 to block its interaction with p53, have shown promising results in NB. These inhibitors attenuate the proliferation of MYCN-expressing NB cells and some of them are being tested in clinical trials; however, the development of resistance, toxicity, MDM2 accumulation, and the need for wild-type p53 make the trials challenging^78^. In addition to the regulation of p53–MDM2, MYCN facilitates an increase in the expression of FAK (focal adhesion kinase), which interacts with p53 and causes its sequestering in the cytoplasm (Figure 2). Interrupting this binding by small molecules or peptides enables p53 to move to the nucleus to induce apoptotic cell death of *in vivo* breast and colon tumors^79^.

Furthermore, MYCN-upregulated MDM2 can similarly bind with another member of the p53 family, tumor suppressor TAp73 (p73 locus encodes two isoforms – tumor suppressor (TAp73) and putative oncogene (ΔNp73)) (Figure 2). MDM2 decreases TAp73 transcription and supports resistance to the treatment^80,81^. It has been discussed that besides regulating p53 and MDM2 levels, MYCN might also directly decrease TAp73 expression and support NB tumor growth^82^. In addition, there are results showing that overexpression of TAp73 can in turn reduce MYCN expression and induce differentiation of NB cell lines, indicating that the balance between TAp73 and MYCN levels can influence the outcome of the NB development and treatment (Fig. 2)^83,84^. These new approaches have led to novel combinatorial therapeutic strategies that simultaneously reduce toxicity and enhance the outcome of the treatment and are being tested in preclinical and clinical trials for NB^75^, melanoma^85^, prostate cancer^86^, and renal cell carcinoma^87^. Although bearing in mind that MYCN has many cellular targets, disrupting its interaction with one of them is probably not enough for successful treatment.

#### BCL-2 family

Other important apoptosis regulators are B-cell lymphoma/leukemia 2 (BCL-2) family proteins, which are divided into two groups: pro-apoptotic and anti-apoptotic proteins. The main anti-apoptotic proteins are BCL-2, BCL-xL, and myeloid cell leukemia (MCL)-1, which prevent outer mitochondrial membrane (OMM) permeabilization by binding and inhibiting pro-apoptotic proteins. Apoptosis-promoting proteins from this family can in turn be divided into two groups: BH-3 only and effector proteins. The pro-apoptotic BH-3 only proteins (Bid and Bim) respond to apoptotic stimuli and inhibit anti-apoptotic BCL-2 proteins or activate the effector proteins (BAK and BCL-2-associated X protein), which form pores in the OMM to induce cytochrome *c* release and apoptosis. The balance between pro- and anti-apoptotic proteins determines the fate of the cells through regulation of the mitochondrial apoptotic pathway^88,89^. As with *p53*, mutations in *BCL-2* are scarce in NB, although dysregulation and increased levels of the *BCL-2* gene are frequent^90–92^. Moreover, in B-cell lymphomas a link between MYC and BCL-2 expression has been described, because overexpression of *MYC* in tumor cells is often found together with rearrangements in the BCL-2 family to support tumor growth and suppress apoptosis^93,94^. Therefore, therapies that change the balance between pro- and anti-apoptotic proteins are promising strategies for tumor treatment.

One possible approach might be using conventional chemotherapeutics together with inhibitors of anti-apoptotic BCL-2 proteins (e.g., ABT-199)^95^, although there have been problems with modest outcome, side effects,^96^ and resistance in relapsed NBs^97^. This is due to the compensatory upregulation of the anti-apoptotic MCL-1 protein that rescues cells from apoptosis. However, when the MCL-1 inhibitor (e.g., A-1210477) is used in combination with ABT-199, successful induction of NB cell death has been demonstrated^98^.

#### Targeting cellular bioenergetics pathways

Considering the key role of mitochondria in various modes of cell death, they might be potential targets for tumor therapy. For instance, many anticancer drugs destabilize mitochondria to induce apoptotic cell death^99^. Rapidly proliferating tumors easily become hypoxic, which is the reason why the majority of tumors change their source of energy from mitochondrial oxidative phosphorylation (OXPHOS) to glycolysis. These cells usually have lowered amount of mitochondria and/or mutations in one or more OXPHOS complexes^100–102^. In contrast, relapsing cancer cells tend to have increased levels of OXPHOS^103–105^. The role of *MYC* overexpression in these processes is to increase the expression of mitochondrial complexes and hence mitochondrial respiration^38^. These metabolic changes help cells to survive in nutrient-deprived environments^106^. Therefore, to eliminate resistant tumor cells, chemotherapeutic drugs could be used in combination with electron transport chain inhibitors, such as the complex I inhibitors metformin^107^ or tamoxifen^108^, to induce leakage of electrons and excessive formation of reactive oxygen species (ROS). In addition, using non-toxic doses of the complex II blockers of the respiratory chain, such as thenoyltrifluoroacetone^109^ or α-tocopheryl succinate^110^ together with harmless doses of cytotoxic drugs, synergistically stimulates the formation of ROS and thereby increases the effectiveness of the therapy on breast cancer and NB cell lines.

**Fig. 3:**
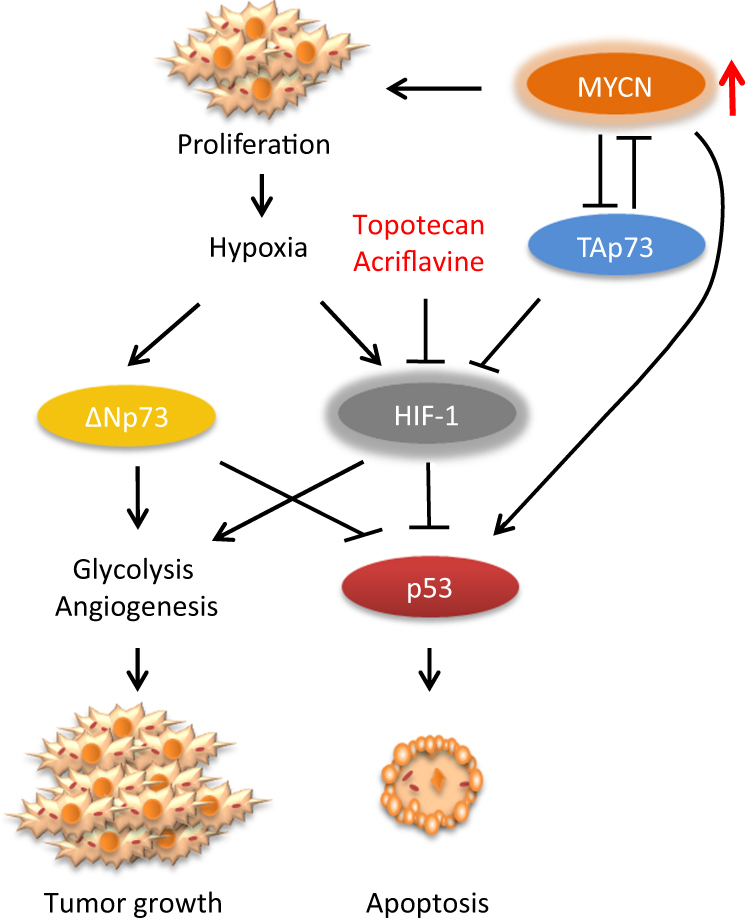
MYCN overexpression facilitates the fast growth of the tumor cells, leading to poor vascularization and hypoxia. This results in the stabilization of transcription factors HIF-1, inhibition of apoptosis, and stimulation of glycolysis, angiogenesis, and rapid tumor growth. In addition, putative oncogene ΔNp73 is stabilized in O_2_-deficient environment and supports vascularization. However, TAp73 regulates degradation of HIF-1 and suppression of vascularization in an oxygen-independent manner. Therefore, considering the importance of HIF-1 in tumor progression, several HIF-1 inhibitors, such as topotecan, phenethyl isothiocyanate, and acriflavine, have shown promising results

Fast growth of the tumor cells and poor vascularization leads to hypoxia, which causes the activation of transcription factors, such as hypoxia-inducible factor 1 (HIF-1), that regulate the hypoxic adaptation (Figure 3)^111–113^. Specifically, HIF-1 regulates developmental and physiological pathways that facilitate O_2_ delivery to the cells or help cells to survive in low O_2_ conditions. HIF-1 is activated in a hypoxic environment that is very common in solid tumors. HIF-1 expression leads to the activation of glycolysis and angiogenesis, and correlates with aggressive tumors and poor outcome. HIF-1 is a heterodimer consisting of the O_2_-regulated HIF-1α subunit and a constantly expressed HIF-1β subunit^114,115^. HIF-1α becomes stabile in a low O_2_ environment and binds with HIF-1β to form an active HIF-1 complex that has both anti- and pro-apoptotic effects^116,117^. For instance, severe and continuous hypoxia will result in HIF-1 activation, p53 expression, and apoptosis. On the other hand, simultaneous stabilization of HIF-1 with activation of the PI3K/Akt pathway, survivin, glycolytic enzymes, p21, and/or erythropoietin can inhibit apoptosis and support NB tumor growth^118,119^.

Furthermore, recent data suggest that the aforementioned TAp73 also regulates the degradation of HIF-1 and the suppression of vascularization in an oxygen-independent manner (Fig. 3)^120,121^. Therefore, loss of TAp73 activity in MYCN-overexpressed tumors can be associated with increased HIF-1 activity and thereby the stimulation of angiogenesis in tumor cells^120,122^. Another isoform of p73, NH_2_ terminally truncated putative oncogene ΔNp73, is also involved in angiogenesis regulation (Fig. 3). In tumor cell lines, ΔNp73 is stabilized in O_2_-deficient conditions and activates vascularization via vascular endothelial growth factor A expression^121^, indicating that cellular response to hypoxic conditions and HIF-1 activity is tightly regulated by MYCN and p53 family proteins. Moreover, HIF-1 activity is also associated with low responsiveness to differentiation therapy and the downregulation of HIF-1 can improve the outcome of the NB treatment^123^. Therefore, taking into account the importance of HIF-1 in NB tumor progression, the search for its inhibitors, such as topotecan^124^ and acriflavine^125^, is a promising strategy. Several of these have already been shown to improve the effects of anti-angiogenic drugs *in vivo*.

Cancer cells modify their metabolism to support their constant proliferation. Adjustments in cancer cells’ metabolism result in excessive glycolytic activity to produce ATP, the Warburg effect, to support rapid cell proliferation. These changes are also seen in aerobic conditions, even though glycolysis generates less ATP than OXPHOS^126,127^. This decrease in oxygen demand helps tumor cells to survive in hypoxic conditions and continue proliferation due to excessive glycolytic activity^128^. Such a drastic metabolic change is attained by the activity of various oncogenes and regulatory proteins, such as *MYC* and HIF-1 (ref. 129,130).

Oncogenic *MYC* upregulates glucose import (e.g., GLUT1), glycolytic enzymes (e.g., hexokinase 2 (HK2) and PDK1), and mitochondrial biogenesis, thereby ensuring metabolic intermediates that support cell growth^131,132^. Elevated glucose transport into the cells and glycolysis itself can be targeted for cancer cell-specific therapy^133,134^. For example, glucose analog 2-DG (2-deoxy-d-glucose) that is phosphorylated by HK2 cannot be metabolized further and accumulates in the cell, leading to the inhibition of glycolysis and tumor growth ^135–138^. This approach has been successful in several NB cell lines^139^ and also in xenograft models,^140^ regardless of their *MYCN* status, indicating its potential for clinical significance. Furthermore, the clinical efficacy of 2-DG is enhanced when combined with cytotoxic drugs in breast^141^, head and neck^142^, and ovarian^143^ cancer cell lines.

Another hexokinase inhibitor lonidamine was under clinical trials and revealed promising results in combination therapy for ovarian cancer clinical trial^144^ and NB cell lines^145^. Furthermore, HK inhibitor 3-bromopyruvate (3-BrPA) effectively reduces cell growth of leukemia^146^, breast^147^, and colon^146^ cancer cells without any significant toxicity or recurrence^146,147^. It has been efficient when used alone or in combination with other inhibitors (e.g., rapamycin^148,149^) or cytotoxic drugs (e.g., platinum-based agents^150^ and doxorubicin^151^) for NB, leukemia, breast, lymphatic, colon, and hepatic cancers. There is also a modified version of 3-BrPA named 3-bromo-2-oxopropionate-1-propyl ester, which is a cell-permeable ester that has a strong effect on GLUT1- and MKI67-expressing NB cells, but is less damaging for normal cells^152^. In addition to HK inhibitors, small-molecule PDK (pyruvate dehydrogenase kinase) inhibitors, such as dichloroacetate (DCA)^153,154^, or the downregulation of lactate dehydrogenase A (LDHA)^155^ can also be used to reverse the glycolytic shift by directing pyruvate into mitochondria, to restore the characteristic phenotype of non-malignant cells. For example, DCA has successfully reduced lactate production, proliferation rate, cell viability, and increased respiration in NB cell lines^156,157^. In addition, LDHA inhibitor FX11 has successfully inhibited aerobic glycolysis and growth of NB cell lines^158^.

Besides increased glucose metabolism, many tumors, and especially NB, show signs of glutamine dependency^159^. Glutamine regulates cellular energetics, redox state, amino acid production, cell signaling, and nucleotide synthesis^160,161^. Therefore, glutamine addiction helps cancer cells to acquire substrates for rapid proliferation and to survive better in complex environments. In tumors, stimulation of glutaminolysis in low glucose and oxygen conditions is mainly induced by MYC, whereas *MYC* knockdown results in reduced glutamine metabolism in glioblastoma cell line^162^. Thus, removal of glutamine should lead to the death of addicted cells, whereas oxaloacetate, pyruvate, and α-ketoglutarate can rescue cells from dying, suggesting that *MYC*-driven glutamine metabolism is a major carbon source for the tricarboxylic acid cycle^162–165^. Therefore, targeting glutamine metabolism for *MYC*-driven tumors is a promising strategy for cancer therapy.

Glutamine depletion results in activating transcription factor 4 (ATF4)-dependent, but p53-independent, apoptosis as a result of the stimulation of expression of the pro-apoptotic BCL-2 family proteins PUMA and NOXA. Therefore, combinations of ATF4 agonists and glutaminolysis inhibitors have shown the induction of apoptosis and a decrease in NB tumor growth^164^. Inhibitors of glutaminase 1 by small molecules such as 986 (ref. 166) and bis-2-[5-phenylacetamido-1,2,4-thiadiazol-2-yl] ethyl sulfide^167–169^, suppressed cell growth, migration, invasion, and resistance to oxidative stress in MYC-overexpressing tumors. However, *MYCN*-amplified NB cells that predominantly express GLS2 might be less sensitive to these drugs^164,167^. Besides GLS blockers, inhibitors of glutamate dehydrogenases, such as epigallocatechin-3-gallate^170^, or aminotransferases, such as aminooxyacetate^171^, can be used to block subsequent glutamate processing. However, problems with identifying the predominant pathway in specific cancers make it difficult to predict the NB sensitivity to these drugs.

### Autophagy and NB therapy

Autophagy is a catabolic survival mechanism that is activated in somatic cells under metabolic stress, to provide the cell with metabolites and to eliminate damaged organelles, protein aggregates, and infecting organisms. Extensive autophagy can also lead to cell death, but its function is not yet fully understood^172–175^. In many solid tumors, including NB, the outcome of the chemotherapeutic agents is also affected by the cellular stimulation/activation of autophagy, which can lead to unexpected consequences and autophagy-mediated cell survival or death^176^. However, there are ongoing discussions and research to better understand whether extensive activation of autophagy could be used to induce cell death or whether it should be blocked, because it helps cells to survive in extreme environments and therefore support tumor growth.

For example, one of the reasons why previously discussed ALK inhibitors may cause resistance is due to their ability to activate autophagy-mediated cell survival. This can be avoided by using ALK inhibitors together with autophagy inhibitors, such as chloroquine, which have been shown to increase cell death of ALK-positive lung cancer^177,178^ In addition, research on histone deacetylase 10 has shown its role in autophagy-mediated cell survival and poor outcomes in high-risk NB^179^. Moreover, BCL-2, a regulator of apoptosis, also controls and inhibits autophagy, which is why it seems to be one of the key factors and a potential target in balancing autophagy and apoptosis^180^. Therefore, inhibition of autophagy in combination with other apoptosis-inducing drugs is a potential strategy to induce apoptotic cell death of NB cells, especially in resistant tumors^181,182^.

#### Targeting PI3K/AKT/mTOR pathway

The PI3K/AKT/mTOR (mechanistic target of rapamycin) signaling pathway is an important regulator of autophagy. In NB, it correlates with a poor outcome and is shown to be upregulated by constitutively activated *ALK* and *MYCN* genes^183–185^. The PI3K/AKT/mTOR pathway is regulated by the aforementioned RTKs, which are shown to be involved in malignant NB cell transformation, when mutated and/or amplified. Therefore, several inhibitors of RTK and PI3K/AKT/mTOR pathways have also been tested for NB therapy^186,187^. However, there are also problems with resistance, as these inhibitors cause secondary mutations and autophagy activation that supports cell survival^188,189^.

Protein kinase mTOR is considered to be the main inhibitor of autophagy and controller of cellular metabolism^190–192^. Deregulation of mTOR expression is very common in tumor cells and it is targeted in many NB studies, as its inhibition destabilizes MYCN, reduces NB growth, and induces excessive autophagy activation that will result in the stimulation of cell death^36,184,193^. Although clinical benefits from mTOR inhibitors, when used alone, have been modest, their effectiveness for NB in combination therapies is under investigation^194–197^. For example, the mTOR inhibitor temsirolimus (rapamycin analog) has been tested for NB in clinical trials, in combination with standard chemotherapy and monoclonal antibodies (NCT01767194)^195^. In addition, the combination of mTOR inhibitors, such as dactolisib^198^, or INK128 (ref. 199), with ALK inhibitors or other conventional chemotherapeutics has shown the ability of the treatment to overcome drug resistance and to prevent NB tumor growth. Moreover, elevated levels of AKT are also very common in NBs^185^. Studies on combined AKT targeting have shown even more successful results, for example, the combination of AKT inhibitor perifosine and mTOR inhibitor temsirolimus is in clinical testing for pediatric solid tumors (NCT01049841)^200^. Furthermore, AKT inhibitor MK2206 in combination with etoposide or rapamycin has shown promising results in NB cell lines^201^. Taken together, targeting the PI3K/AKT/mTOR pathway and thereby inducing excessive autophagy can be used as a strategy for cancer therapy; however, targeting several pathways simultaneously should be used to avoid resistance to treatment.

### Necroptosis induction in NB therapy

Cellular stress can activate various caspase- and p53-independent forms of cell death in normal and transformed cells. One of them is necroptosis, which is morphologically similar to inflammation and immune response caused by necrosis^202^. It is mediated by necrotic death receptors, their ligands, interferons, Toll-like receptors, and the necrosome complex, consisting of receptor-interacting protein kinases 1/3 (RIPK1/3) and mixed lineage kinase domain-like^203–206^. Necrosome formation induces mitochondrial ROS production and the release of apoptosis-inducing factor, which are thought to be important executors of necroptosis^206,207^. Normal cell survival is supported by the inhibition of apoptosis and necroptosis, where apoptosis induction is suppressed by FLICE-inhibitory protein inhibiting caspase-8 (ref. 208) and necroptosis induction is blocked by caspase-8-mediated cleavage of RIPK1/3 (ref. 209). Therefore, the balance between these proteins will determine whether the cell will survive or die and through which pathway. Thus, it is expected that necroptosis has an important role in several human disorders, such as neurodegenerative and inflammatory diseases^210^. Moreover, necroptotic cell death can be used as a novel approach to modulate antitumor immunity and apoptosis in the treatment of resistant cells^211^.

As many aggressive NBs do not express caspase-8 and are resistant to apoptosis, inducing necroptotic cell death to eliminate these cells is another strategy to increase the efficiency of treatments^212^. One way to trigger necroptosis in NB cells is through the increase of cytoplasmic Ca^2+^ that activates calcium-calmodulin kinase II, which in turn activates RIPK1 (ref. 213). Other agents inducing necroptosis in RIPK3-expressing NB cells are polyphyllin D^214^ and d-gal^215^. On the other hand, many NBs have a decreased expression of caspase-8 and low level of proteins involved in necroptosis, especially in the advanced stages, making them also resistant to necroptosis induction^216^. It is not clear why these genes are downregulated in NB, but epigenetic modifications may be the reason of this outcome. Thus, demethylating drugs and/or histone deacetylase inhibitors^217,218^ can be used to overcome this issue and support the use of necroptosis as a new approach for NB therapy.

### Immunotherapy in NB treatment

Owing to the limitations of current therapies, many immunotherapeutic approaches can be used to induce NB cell death through redirecting the immune system to eliminate the malignant cells and to achieve long-term immunity and protection against relapse. One way is through targeting ALK-positive NBs with antibodies, to inhibit cell growth and induce cytotoxicity^219,220^. Antibodies can also be used to deliver immunotoxins, radioisotopes, liposomes, or nanoparticles^221^. This new method of drug delivery has a high potential for very specific on-the-spot effects on tumor cells, at the same time avoiding toxicity on healthy cells.

This approach is also used for other surface epitopes, because NB is derived from embryonic tissue and it expresses surface antigens that are not widespread in non-embryonic tissues, such as L1-cell adhesion molecule (L1-CAM), GD2/3 (disialoganglioside), and B7H3 (ref. 222–224). These antigens can be used as biomarkers to target advanced and chemotherapy-resistant NB cells with immunotherapeutic antibodies. The described strategy has shown promising results in preclinical and clinical trials with monoclonal antibodies, such as Hu3F8 (ref. 225–228) and dinutuximab^229–232^, on GD2-positive NB tumors. It has been shown that treatment with these antibodies will lead to cytotoxicity mediated by monocytes, macrophages, granulocytes, the complement system, and natural killer (NK) cells. As anti-GD2 antibodies act via cell-mediated cytotoxicity and NK cell reactivity, NB patients with higher immune activity have better outcomes from this treatment.^233–238^ This method seems to be even more effective when used in combination with cytotoxic chemotherapy, cytokines, adoptive NK cell therapy, and 13-*cis-*retinoic acid^232,239–245^. However, there have been problems with treatment efficiency, pain toxicity, and relapse; attempts to eliminate these issues have not yet been fully successful^244^. Another problem with this kind of treatment is that, generally, it does not induce immunological memory and other parts of the immune system should be used to achieve long-term effects.

For instance, there is evidence for “natural immunity” against ALK-positive NB cells. This is due to NB’s peculiarity in presenting ALK peptides on human leukocyte antigen I, which is then recognized by T cells^246,247^. This led to a novel strategy that uses designed and/or activated T cells to induce bio-distributed, long-term, and direct cytotoxicity, which is free of the immunosuppressive influences of the tumor. These designed T cells have a chimeric antigen receptor against GD2, L1-CAM, or ALK, and they have demonstrated safety and no pain toxicity in relapsed NB^248–253^. Another similar approach is to use a peptide vaccine, such as ganglidiximab^254^, made from the tumor proteins, to activate T cells against the NB^255–257^. These strategies are already in clinical trials and demonstrating high efficiency. However, there are several potential drawbacks with these therapies, starting with the low or altered expression of HLA and its co-stimulatory molecules on the cells, complex and expensive standardization processes, and its requirement to use disease compromised immune system^246,258^.

### Spontaneous regression and TrKA pathway

NB is known for its spontaneous regression by differentiation or reactivated apoptosis, which can be considered as a possible strategy for improved therapy^259,260^. Experiments with differentiation supporting vorinostat^261^, a histone deacetylase inhibitor, and didymin^262^, a citrus-derived compound, have resulted in regression of NB in xenograft models and differentiation in relapsed NB^261,262^. There are also several other simple compounds, such as all-*trans* retinoic acid^263–269^, nitric oxide^270^, and phenylacetate^267^ that trigger the induction of differentiation and inhibition of NB growth by inducing the expression of neural differentiation genes. However, this mechanism is not clear, but there is evidence that NB spontaneous regression caused by retinoids is associated with increased expression of tropomyosin receptor kinase A (TrkA) receptors^269,271^.

Furthermore, spontaneous regression of NB is correlated with high expression of TrkA and its ligand nerve growth factor (NGF), which protects cells from apoptosis and directs them to differentiation, whereas NGF alone promotes apoptosis^272–277^. Therefore, changing the balance between TrkA and NGF expression can be used for the activation of NB differentiation and apoptosis. For example, re-expression of exogenous TrkA in NB cells guides cells to NGF-induced differentiation.^274,277–279^ Apoptotic cell death can be induced by TrkA inhibitors, like K252a (ref. 280), and GTx-186 (ref. 281) or by downregulating TrkA with miRNA-92a (ref. 282), however, these strategies are not yet clinically tested for NB. NGF can also sensitize TrkA-expressing cells for TRAIL-induced apoptosis and this effect can be further increased by using inhibitors of NF-*κ*B and/or Mcl-1 (ref. 283). However, this approach may work better for the primary NB, but not relapsed NB, which often has mutations in this regulatory pathway.

Another Trk family protein kinase is TrkB, whose expression is correlated with poor NB prognosis and *MYCN* amplification. For example, TrkB ligands, such as BDNF and NT-4/5, are distributed via autocrine or paracrine signaling to support overall NB viability, drug resistance, and angiogenesis of TrkB-positive tumors^284–286^. Therefore, targeting TrkB may reduce the malignancy of NB with dysregulated TrkB, which can be achieved by the TrkB inhibitors GNF-4256 (ref. 287) or AZD6918 (ref. 288), which have shown promising results alone and in combination in a xenograft mouse model.

Moreover, expression of a homeobox gene *HOXC9* is associated with a favorable prognostic outcome and is known as a marker of spontaneous regression in infant NBs, whereas its downregulation is present in advanced-stage NBs. Therefore, re-expression of *HOXC9* can be used to induce NB regression or activation of apoptotic cell death in NB cell lines^289,290^. Based on all of the aforementioned information on spontaneous regression in NB, it is not clear how it is regulated. Regression seems to be as complex mechanism as all the other cellular pathways and it can include a variety of cross-talking cell death mechanisms.

## Conclusion

Therapeutics inducing different modes of cell death, mainly apoptosis, have been proved to be successful, but sometimes they demonstrate a modest efficiency and side effects. The main problem with stimulating apoptosis in tumor cells is their ability to compensate for pro-apoptotic signals via upregulating anti-apoptotic agents. Therefore, searching new strategies is crucial to achieve improved outcome of NB therapy. One way to enhance the treatment is to understand better the genetic and metabolic background of NB. This in turn can be used for more specific and even personalized therapy, thereby improving the outcome of the treatment. Moreover, recent developments in NB treatment are directed towards combined therapies that target many pathways, not just different sites of one pathway. Another promising and clinically tested approach is immunotherapy, which can be used to induce NB cell death through redirecting the immune system to eliminate the malignant cells and to achieve long-term immunity and avoid relapse. However, there are several potential drawbacks, starting with the requirement to use healthy and functional immune system, as well as difficult and expensive standardization processes. Thus, there is no easy way to overcome this complex and heterogeneous disease, but step-by-step improvements are bringing us closer to prolonged survival and gain in life quality.
